# Strategy to Decrease the Angle Measurement Error Introduced by the Use of Circular Grating in Dynamic Torque Calibration

**DOI:** 10.3390/s21227599

**Published:** 2021-11-16

**Authors:** Yongbin Du, Feng Yuan, Zongze Jiang, Kai Li, Shuiwang Yang, Qingbai Zhang, Yinghui Zhang, Hongliang Zhao, Zhaorui Li, Shunli Wang

**Affiliations:** 1 School of Instrumentation Science and Engineering, Harbin Institute of Technology, Harbin 150001, China; duyongbinl@163.com (Y.D.); jiangzz@hit.edu.cn (Z.J.); likaihit@hit.edu.cn (K.L.); 19B901047@stu.hit.edu.cn (Y.Z.); zhaohongliang360@163.com (H.Z.); 20B901054@stu.hit.edu.cn (Z.L.); 16b901019@stu.hit.edu.cn (S.W.); 2 Beijing Zhenxing Institute of Metrology and Measurement, Beijing 100074, China; suiwang_y@163.com (S.Y.); boboterry8279@163.com (Q.Z.)

**Keywords:** dynamic torque calibration, circular grating, eccentricity error, inclination error, error compensation

## Abstract

A circular grating angle encoder is a key component in the dynamic torque calibration system. To improve the accuracy of an angle measurement, in this paper, the source of the angle measurement error of the circular grating is analyzed; an eccentricity error model and an inclination error model are proposed, respectively; further, these two models are combined to establish a total error model. Through the simulation study with the models, the conditions, in which the eccentricity error or inclination error can be ignored, are discussed. The calibration and compensation methods of the angle measurement error are given, and a progressive error compensation function which integrates the first harmonic fitting and the second harmonic fitting is obtained. An experiment is performed to verify the proposed calibration and compensation methods. The peak-to-peak value of the compensated angle measurement error of the single reading head can be reduced by about 93.76%, which approximates to the error of the mean value of the double reading heads. The experimental results show that the error calibration and compensation method based on the proposed error model can effectively compensate the angle measurement error of the circular grating with a single reading head, and obtain a high-precision measurement angle.

## 1. Introduction

Torque transducers are widely used in rotating machinery [1,2], such as engines, motors, generators, propellers, etc. The calibration, measurement, and analysis of torque transducers is the key to ensure the normal and safe operation of this equipment [3]. However, the calibration of torque transducers is still in the laboratory static calibration stage, and the research on dynamic calibration models and calibration method is not very extensive. Thomas [4] carried out a sinusoidal torque calibration using the laser interferometric method, and initially realized the calibration of a sinusoidal excitation torque with a maximum frequency of 100 Hz and amplitude of 100 N·m. Then, Georg et al. [5] realized the calibration of the torque sensor under a dynamic torque and rotation conditions, respectively. Zhang et al. [6] developed a dynamic torque calibration device with a calibration range of 0.1–200 N·m and a maximum frequency of 100 Hz.

In the dynamic torque calibration system, the circular grating angle encoder (referred to as “circular grating” for short) is a common angle measuring component. The application of the high-precision and high-resolution circular grating can improve the accuracy of the torque calibration results. The error of the circular grating angle measurement can be introduced by different conditions, such as the eccentricity of the rotating shafting [7] (referred to as “eccentricity error” for short), the inclination between the axis of rotation and the geometric axis of the rotating shafting [8,9] (abbreviated as “inclination error”), the axial movement of the rotating shafting, the roundness of the radial section of the rotating shafting [10,11], and the circular grating itself [12], etc.

The methods to improve the angle measurement accuracy of circular gratings proposed in previous studies can be summarized as increasing the number of reading heads and using a compensation algorithm. Scholars have conducted a lot of research on the angle measurement of multi-reading heads of circular grating [13,14,15,16,17,18]. Some important studies are listed below. Zhang [13] proved the error elimination principle of the multi-reading heads structure by using the harmonic analysis method. Ralf et al. [14] developed a self-calibration method for the fast and precise in situ calibration of angle encoders without recourse to external reference standards. It depended on the proper geometric arrangement of multiple reading heads, and the use of an algorithm based on Fourier transform to analyze the measurement difference of double heads in order to recover the indexing error of the grating. Liu et al. [15] developed an optimization-based arrangement method for the self-calibration of angle encoders. Ren et al. [16] analyzed the error sources affecting the angle measurement accuracy, and proposed the compensation method of multi-reading heads reading averaging. Based on the Back Propagation neural network, Xue et al. [17] established a model that can compensate the error over the whole circumference. Lou et al. [18] proposed a novel self-calibration method for five degrees-of-freedom error motions of rotary tables, and they installed two encoders with multiple reading heads on the spindle’s different positions to measure rotation angles. The above-mentioned studies all use two or more reading heads to compensate the angle measurement error of the circular grating, but they have not analyzed the specific angle measurement error model of the single reading head. Moreover, when only increasing the number of reading heads without algorithm compensation, the accuracy of the angle measurement is mostly not enough. Therefore, they all provided different error compensation algorithms to improve the accuracy of the angle measurement. However, many commercial grating encoders are equipped with only one reading head, and increasing the number of reading heads would, inevitably, increase the cost of the project. It would, inevitably, increase the error items caused by the installation of the reading heads, and increase the complexity of the error analysis. Furthermore, for some special applications, such as in article [19], the angle measuring system is required to be as light as possible, and a single reading head is usually used. In addition, when using semi-circular gratings and circular arc gratings for an angle measurement, only a single reading head can be used instead of multi-reading heads.

In order to improve the angle measurement accuracy of circular gratings with a single reading head, researchers have conducted a lot of work on the compensation algorithm [7,8,20,21,22,23,24,25,26,27,28,29,30]. Chen et al. [7,8], respectively, derived the eccentricity error model and the inclination error model of the circular grating angle measurement based on the Moiré fringe equations. However, the above research did not consider the influence of the eccentricity error phase and inclination error phase, as well as the interaction between them. Ralf et al. [20] proposed an in-depth treatment of the use of the Fourier approach, including transfer functions for the calibration of angle encoders. Li et al. [21] analyzed the angle measurement error distribution characteristics of the circular grating encoder, and established a method to obtain the angle error compensation value through the uncertainty calculation based on the Monte Carlo method (MCM). Deng et al. [22] presented a method based on the adaptive differential evolution Fourier neural network (ADE-FNN). Mark et al. [23] established a simple method for a high-precision rotary angle encoder calibration for long-range angular errors. Cai et al. [24] proposed a novel error compensation method based on the empirical mode decomposition (EMD) method. Jia et al. [26] established a method based on the Fourier expansion-back propagation (BP) neural network optimized by the genetic algorithm (FE-GABPNN). Gao et al. [27] designed an angle compensation scheme based on Fourier fitting. Different novel error compensation algorithms are proposed in the above studies, but the error model is not discussed in detail. Zheng et al. [28] discussed the influence of the circular grating eccentricity error on the measurement accuracy of the articulated arm coordinate measuring machine, proposed an error compensation parameter method, and established a six-circular grating eccentricity error compensation model. Yu et al. [19] presented an eccentricity error compensation method based on the calibration experiment using a single reading head. The above studies only derive the corresponding eccentricity error model, but do not fully consider the combined influence of the eccentricity error and inclination error. Based on the Abbe principle, Li et al. [29] used a new method to analyze the angular positioning error of the rotary table by using a circular grating with multi-reading heads. Based on compressed sensing and sparsity decomposition, Chen et al. [30] proposed a novel method to improve the angle measurement accuracy of circular grating. Although the above research gave the corresponding eccentricity error model and inclination error model, they did not consider the phase relationship between them. Moreover, when analyzing the inclination error model, the eccentricity caused by the inclination error was not considered. In summary, most of the existing research on the angle measurement of the circular grating with a single reading head is only in the compensation algorithm. In terms of the error model analysis, there is more research on the eccentric error model, and less on the inclination error, and especially less on the relationship between them.

The correctness of the angle measurement error model of the circular grating directly affects the compensation accuracy of the error compensation method. The self-error of circular grating and the error caused by roundness can be reduce to a negligible degree by using the high-precision circular grating. Because the axial movement of the shaft is perpendicular to the measurement plane of the reading head, the axial movement error is orthogonal to the rotation angle, and generally does not affect the angle measurement error of the circular grating. Most of the current models of the angle measurement error of the circular grating do not consider the inclination error, or think that the inclination error is negligible relative to the eccentricity error. In fact, the influence of the inclination error on the angle measurement is also very obvious and, in some cases, it is consistent with the influence of the eccentricity error. Therefore, when analyzing the angle measurement error of the circular grating, the inclination angle error should not be ignored all the time.

The contributions of this paper are as follows: (1) After analyzing the sources of errors, the eccentricity error model and the inclination error model are, respectively, proposed. Further, a total error model combining the two models is established. (2) According to the characteristics in each model, the applicable conditions and approximate simplified application formulas of each model are given. Through the simulation study with the models, the conditions, in which the eccentricity error or inclination error can be ignored, are discussed. (3) The calibration and compensation methods of the angle measurement error are given, and the progressive error compensation function which integrates the first harmonic fitting and the second harmonic fitting is obtained. Then, an experiment is performed to verify the proposed calibration and compensation methods. The angle measurement accuracy after the compensation of the single reading head is consistent with that acquired with the double reading heads. (4) The angle measurement error calibration function is applied to the dynamic torque calibration system, and the simulation results show that this method can improve the accuracy of the dynamic torque calibration system.

The structure of the article is introduced as follows: The dynamic torque calibration system is briefly introduced in the next section. In Section 3, the eccentricity error model and the inclination error model of the angle measurement error of the circular grating are, respectively, derived, and the comprehensive angle measurement error model is given. Then, different simulations are executed based on the three error models, and their results are compared. Section 4 introduces the error calibration experiment and proposes the error compensation method. Section 5 discusses the results of the calibration experiments. The conclusions are presented in Section 6.

## 2. Dynamic Torque Calibration System

Without considering the friction damping, the torque *T* of the rotating shafting can be expressed as the following formula.

(1) $$\;T=J\cdot \ddot{\alpha },$$

where *J* is the moment of inertia, $\;\ddot{\alpha }$ is the angular acceleration.

**Figure 1:**
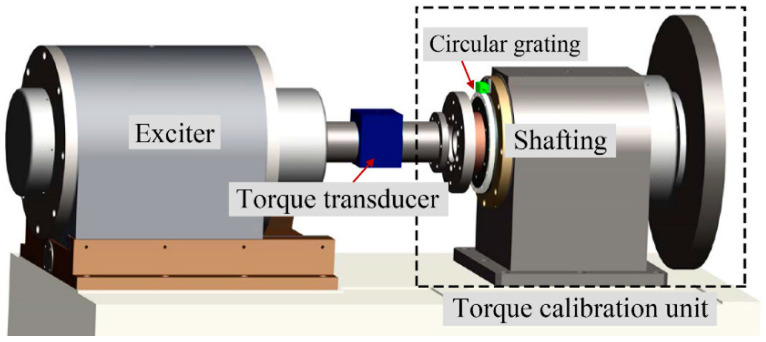
Schematic diagram of the dynamic torque calibration system.

In Equation (1), the dynamic torque is directly traceable to the time, angle, and mass [6]. Therefore, a dynamic torque calibration system was established based on this principle, as shown in Figure 1.

The dynamic torque calibration system mainly included an exciter and a torque calibration unit. The calibrated torque transducer was installed between the exciter and the torque calibration unit. In order to realize the calibration of dynamic torque, we first controlled the exciter to generate a varying torque. Because there was a large error between the real value and the preset value of the torque generated by the exciter, we needed to use the torque calibration unit to accurately measure the actual dynamic torque of the shafting. During excitation, the generated torque was transferred via the torque transducer onto the torque calibration unit to generate the angular acceleration of its shafting. By measuring the angular acceleration and the moment of inertia of the shafting, the dynamic torque could be accurately determined according to Equation (1) [4]. Then, the dynamic torque value measured by the dynamic torque calibration unit was compared with the value measured synchronously of the torque transducer to realize the dynamic calibration for the torque transducer.

As a dynamic torque calibration system, the accurate dynamic torque value had to first be obtained before it could be compared with the measured value of the torque transducer. Therefore, it was required to accurately measure the moment of inertia *J* and angular acceleration $\;\ddot{\alpha }$ of the shafting. The moment of inertia *J* of the shafting of the torque calibration unit was constant in a single experiment and could be measured in advance by the torsional pendulum method [31]. In the calibration experiment, the rotation angle *α* of the shafting was dynamically measured by a circular grating. The angular acceleration$\;\ddot{\alpha }$ could be obtained by twice differentiating the measured angle *α*. Then, substituting *J* and $\;\ddot{\alpha }$ into Equation (1), the dynamic torque could be obtained. Finally, the dynamic torque results were used to calibrate the torque transducer. In order to obtain a higher calibration accuracy, the dynamic torque calibration system was equipped with a high-precision circular grating for the angle measurement, and the error compensation was performed on the measured angle. In the following sections, the error model and compensation method of the circular grating angle measurement was discussed in detail.

## 3. Establishment and Simulation Analysis of Angle Measurement Error Model

### 3.1. Angle Measurement Error Modeling

#### 3.1.1. Eccentricity Error Modeling

**Figure 2:**
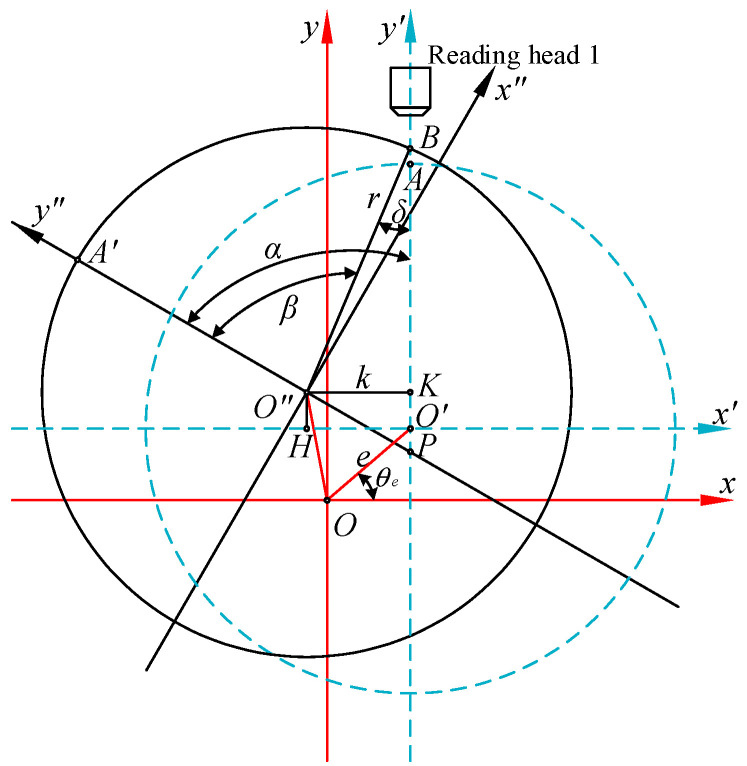
Schematic diagram of the eccentricity error of angle measurement with a single reading head.

Without considering the inclination error, the eccentricity error of the circular grating with a single reading head is shown in Figure 2.

In Figure 2, the blue dotted circle indicates the circular grating radial section where the measuring point of reading head one was located (referred to as “reading section” for short). *r* is the radius of the radial section. The rotation center *O* and the geometric center *O*′ of the reading section generally did not coincide. The distance between these two points is the eccentricity *e*. According to the position of the measuring point of reading head one, two two-dimensional rectangular coordinate systems were established on the reading section, namely, the fixed coordinate system (FCS) *xOy* and the rotating coordinate system (RCS) *x′O′y′*. When the reading section rotated counterclockwise from the position of the blue dotted circle to the position of the black solid circle, *α* was the actual rotation angle of the shafting. The rotating coordinate system *x′O′y′* rotated counterclockwise around the origin *O* to the coordinate system *x″O″y″* position. The measuring point of reading head one changed from *A* to *B*. *β* is the measured value of reading head one. *δ* is the eccentricity error. Evidently, *δ* = *β* − *α*. The coordinates of each point before and after rotation in Figure 2 are shown in Table 1.

According to the two-dimensional coordinate transformation matrix, we had:

(2) $$\left\{ \begin{array}{l} x_{O\prime \prime }=x_{O\prime }\cos\alpha +y_{O\prime }\sin\alpha \\ y_{O\prime \prime }=-x_{O\prime }\sin\alpha +y_{O\prime }\cos\alpha \end{array}\right.$$

In Figure 2, *O″K* is perpendicular to the *y′*-axis. Then,

(3) $$k=x_{O\prime \prime }-x_{O\prime }=y_{O\prime }\sin\alpha -x_{O\prime }(1-\cos\alpha )$$

Let ∠O′Ox be *θ_e_*, which is called the eccentricity angle, so:

(4) $$\left\{ \begin{array}{l} x_{O\prime }=e\cos\theta e\\ y_{O\prime }=e\sin\theta e \end{array}\right.$$

Substituting Equation (4) into Equation (3), we obtained:

(5) k=e[cos(α−θe)−cosθe]

In Δ*O″KB*, the length of side *O″B* is equal to *r*, then:

(6) $$\sin\delta =\frac{k}{r}=\frac{e}{r}\left[\cos(\alpha -\theta e)-\cos\theta e\right]$$

When the value of *e* was much smaller than *r*, we had:

(7) $$\delta \approx \frac{e}{r}\left[\cos(\alpha -\theta e)-\cos\theta e\right]$$

Equation (7) shows that the eccentricity error was related not only to the eccentricity *e*, but also to the eccentricity angle *θ_e_*. *θ_e_* has four special values:

1. When *θ_e_* = ±π/2, $x_{O\prime }=0$ and $y_{O\prime }=\pm e$. In this case, the initial *y′*-axis of the RCS coincides with the *y*-axis of the FCS. Then, Equation (7) can be simplified to:
  (8) $$\delta =\pm \frac{e}{r}\sin\alpha$$
2. When *θ_e_* = 0 or *θ_e_* = π, $y_{O\prime }=0$, $x_{O\prime }=\pm e$. In this case, the initial *x′*-axis of the RCS coincides with the *x*-axis of the FCS. Then, Equation (7) can be simplified to:
  (9) $$\delta =\pm \frac{e}{r}(1-\cos\alpha )$$

#### 3.1.2. Inclination Error Modeling

**Figure 3:**
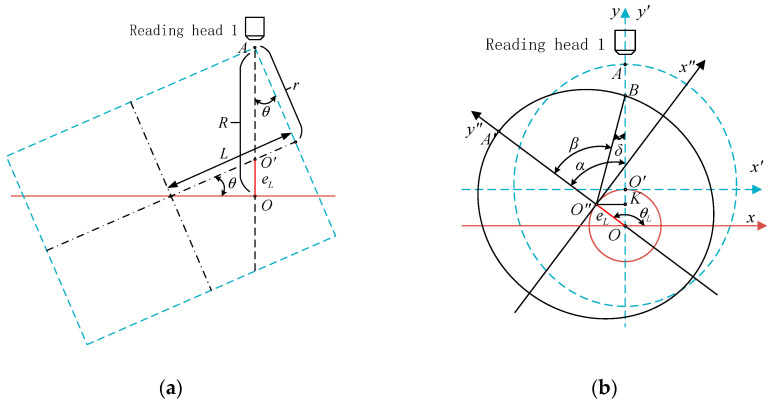
Schematic diagram of the inclination error of angle measurement with a single reading head. (**a**) Axial section of the shafting; (**b**) reading section of the shafting.

Without considering the eccentricity error, the inclination error of the circular grating with a single reading head is shown in Figure 3.

In Figure 3a, *θ* is the included angle between the rotation axis and the geometric axis of the shafting, which is called the inclination angle. *L* is the distance from the spatial intersection of the rotation axis and the geometric axis of the shafting to the radial section of the circular grating. *r* is the outer diameter of the circular grating.

In Figure 3b, *O* is the rotation center, and the reading section rotates around *O*. The red circle represents the trajectory of the geometric center of the reading section. When the rotating coordinate system *x*′*O*′*y*′ rotated counterclockwise around the origin *O* to the coordinate system *x*″*O*″*y*″ position, the geometric center of the reading section rotated from *O*′ to *O*″ at an angle of *α*.

In the reading section, the maximum distance between measuring point *A* of reading head one and the rotation center *O* was:

(10) max(R)=L⋅sinθ+r⋅cosθ

The reading section was an ellipse, and its major axis *a* and minor axis *b* were, respectively:

(11) $$\left\{ \begin{array}{l} a=\frac{r}{\cos\theta }\\ b=r \end{array}\right.$$

Because the value of *θ* is usually very small, it was approximately considered that the reading cross-section was circular (the radius was still equal to *r*) to simplify the calculation. Then, the reading section of the shafting at the inclination was similar to that at the eccentricity. Therefore, the distance from the center of the ellipse to the rotation axis was the eccentricity caused by the inclination, which was:

(12) $$eL=\max(R)-a=L\cdot \sin\theta +r(\cos\theta -\frac{1}{\cos\theta })$$

According to Equation (6), the inclination error was:

(13) $$\delta =\arcsin\left(\left[\frac{L}{r}\sin\theta +(\cos\theta -\frac{1}{\cos\theta })\right]\left[\cos(\alpha -\theta L)-\cos\theta L\right]\right)$$

Because $\cos\theta -\frac{1}{\cos\theta }=\tan\theta \sin\theta \ll \frac{L}{r}\sin\theta$, it was rounded off during calculation. Therefore, Equation (13) could be approximately simplified to:

(14) $$\delta \approx \frac{L}{r}\sin\theta \left[\cos(\alpha -\theta L)-\cos\theta L\right]$$

#### 3.1.3. Total Error Model

When the eccentricity error and the inclination error coexist, the total error combining the two factors is:

*δ* = *δ_e_* + *δ_θ_* (15)

where *δ_e_* is the eccentricity error and *δ_θ_* is the inclination error. According to Equation (7) and Equation (14), we obtained:

(16) $$\delta =\frac{e}{r}\left[\cos(\alpha -\theta e)-\cos\theta e\right]+\frac{L}{r}\sin\theta \left[\cos(\alpha -\theta L)-\cos\theta L\right]$$

When *θ_L_* = *θ_e_*, we had:

(17) $$\delta =\frac{\left(e+L\sin\theta \right)}{r}\left[\cos(\alpha -\theta e)-\cos\theta e\right]$$

The total error increased due to the superposition of the eccentricity error and the inclination error. This condition should be avoided in the experiment, which reduced the accuracy of angle measurement.

When *θ_L_* = *θ_e_* + *π*, we had:

(18) $$\delta =\frac{\left(e-L\sin\theta \right)}{r}\left[\cos(\alpha -\theta e)-\cos\theta e\right]$$

In this case, the two errors could be partially offset. In the experiment, the shafting should work under this condition as much as possible to improve the angle measurement accuracy.

Let *θ_L_* = *θ_e_* + *θ_eL_* and substitute it into Equation (16) to obtain:

(19) $$\delta =-\frac{2}{r}\sin\frac{\alpha }{2}\left[\left(e+L\sin\theta \cos\theta eL)\sin(\frac{\alpha }{2}-\theta e)-(L\sin\theta \sin\theta eL)\cos(\frac{\alpha }{2}-\theta e\right)\right]$$

Let C=e+LsinθcosθeL, D=LsinθsinθeL, $\cos\varphi =\frac{C}{\sqrt{C^{2}+D^{2}}}$ and $\sin\varphi =\frac{C}{\sqrt{C^{2}+D^{2}}}$. Then, we obtained:

(20) $$\delta =-\frac{1}{r}\sqrt{C^{2}+D^{2}}\cos(\theta e+\varphi )+\frac{1}{r}\sqrt{C^{2}+D^{2}}\cos(\alpha -\theta e-\varphi )$$

Let $\delta_{0}=-\frac{1}{r}\sqrt{C^{2}+D^{2}}\cos(\theta e+\varphi )$, $C\delta =\frac{1}{r}\sqrt{C^{2}+D^{2}}$, $\varphi_{0}=\frac{\pi }{2}-\theta e-\varphi$. Then,

(21) $$\delta =\delta_{0}+C_{\delta }\sin(\alpha +\varphi_{0})$$

Error *δ* is a first harmonic function. Then, we could calibrate the measurement angle by fitting the error data to the first harmonic, and provide an error compensation curve. Moreover, after determining the eccentricity parameters (including *e* and *θ_e_*) and the inclination parameters (including *L* and *θ_L_*), we could compensate for the measurement value of the single reading head to obtain a higher measurement accuracy.

### 3.2. Simulation Comparison of Various Models of Angle Measurement Error

#### 3.2.1. Simulation Comparison between the Eccentricity Error Model and the Inclination Error Model

The purpose of the simulation in this section was to analyze the relationship between the models given in Section 3.1. Therefore, the assumed values of these parameters in the model could be arbitrary values, which would not affect the simulation conclusions. However, during the model simulation analysis, we should also consider the actual situation. The radius of a commonly used circular grating is about 100 mm, so let *r* = 100 mm. Because the length of the shafting in the experiment was 71 mm, we assumed that the spatial intersection position of the rotation axis and the geometric axis was just in the middle of the shafting when the shafting rotation produced an inclination angle; therefore, let *L* = 35.5 mm. Because the machining accuracy of the rotating shaft and the bearing was were very small when the shafting rotated, generally *e* ≤ 0.1 mm, *θ* ≤ 0.1°. For ease of analysis, we set *e* and *θ* to be larger values; therefore, let *e* = 0.1 mm, *θ* = 0.1°. In this way, the angle measurement errors caused by them would be larger, which was helpful for us to observe the characteristics of these errors and draw correct conclusions.

Let *r* = 100 mm and *e* = 0.1 mm. The shafting rotated one turn, and the value of *α* was from 0° to 360°. The angle measurement error data calculated by using the proposed eccentricity error model (Equation (7)) are shown in Table 2.

**Figure 4:**
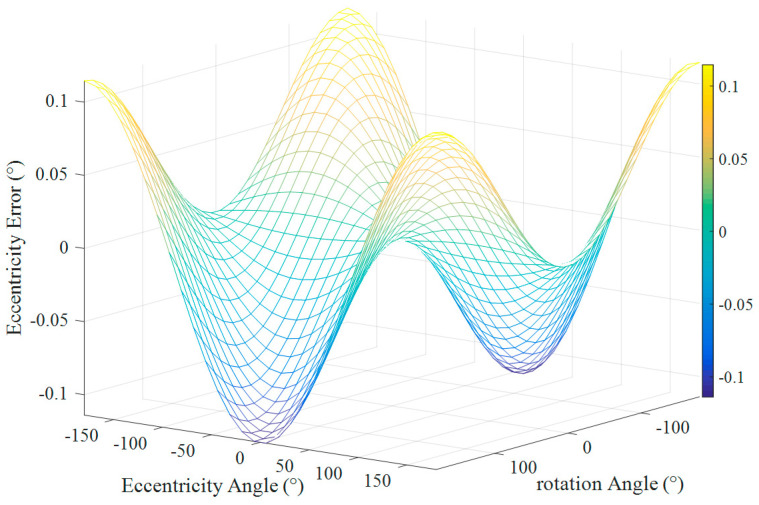
The eccentricity error *δ_e_* varied with the eccentric angle *θ_e_* (when *e* = 0.1 mm).

The relationship among eccentricity error *δ_e_*, eccentricity angle *θ_e,_* and rotation angle *α* is shown in Figure 4.

As shown in Figure 4, when the eccentricity *e* was the same and the eccentricity angle *θ_e_* was different, the eccentricity error results obtained by Equation (4) were also different. It showed that the eccentricity angle was an important parameter for the eccentricity error compensation. Only when the eccentricity angle was determined could the initial position of the rotation be determined, and the measurement result could be compensated.

Let *r* = 100 mm, *L* = 35.5 mm, *θ* = 0.1°, and *θ_L_* = *θ_e_* + 30°. The shafting rotated one turn, and the value of *α* was from 0° to 360°. The angle measurement error data calculated by using the proposed inclination error model (Equation (14)) are shown in Table 3.

**Figure 5:**
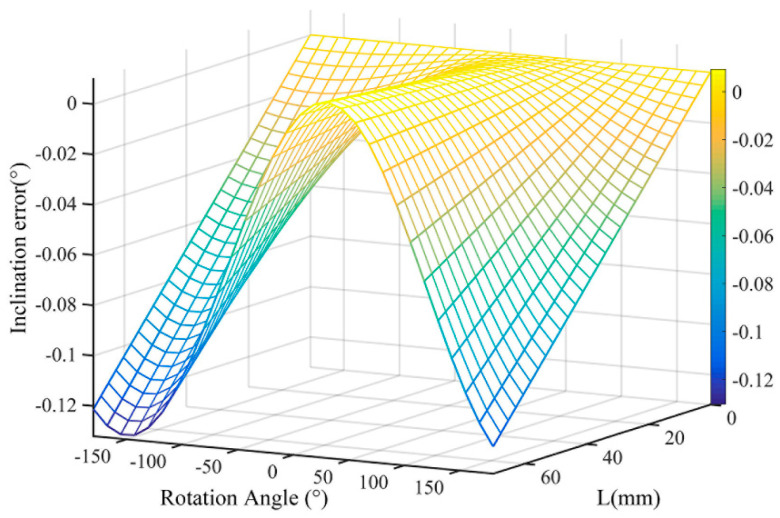
The inclination error varied with *L* (when *θ* = 0.1°).

Let *r* = 100 mm, *θ* = 0.1°, and *θ_L_* = 30°. The shafting rotated one turn, and the value of *α* was from 0° to 360°. The relationship among the inclination error *δ_L_*, *L*, and the rotation angle *α* is shown in Figure 5.

From the comparison of the data in Table 2 and Table 3, it can be seen that the inclination error and the eccentricity error were in the same order of magnitude. The same conclusion can be drawn by comparing Figure 4 and Figure 5. Therefore, when analyzing the angle measurement error of the circular grating, the inclination angle error is also a very important factor and cannot be ignored all the time.

#### 3.2.2. Total Error Simulation

Let *r* = 100 mm, *L* = 35.5 mm, *θ* = 0.1°, and *θ_L_* = *θ_e_* + 30°. The shafting rotated one turn, and the value of *α* was from 0° to 360°. The angle measurement error data calculated by using the proposed total error model (Equation (16)) are shown in Table 4.

**Figure 6:**
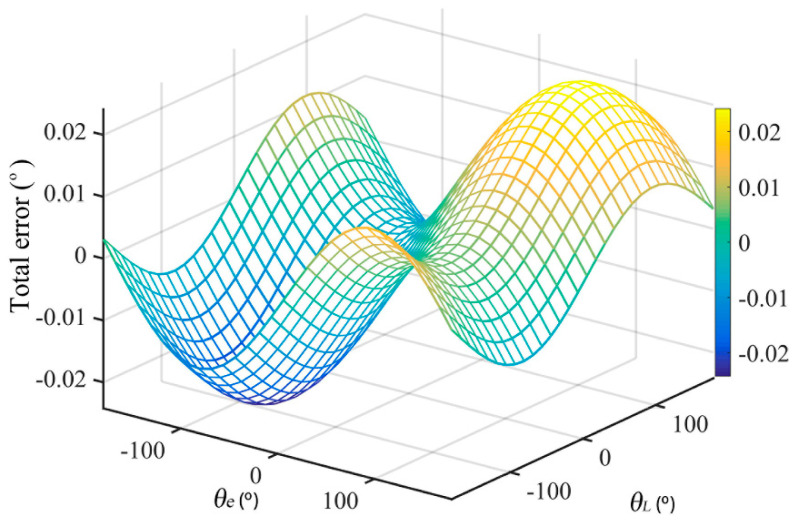
The total error *δ* varied with *θ_e_* and *θ_L_*.

Let *r* = 100 mm, *L* = 35.5 mm, *θ* = 0.1°, *α =* 15°, θe∈[−π,π], and θL∈[−π,π]. The relationship among the total error *δ*, *θ_e_*, and *θ_L_* is shown in Figure 6.

Let $\lambda =\frac{e}{L\sin\theta }$, which is the amplitude ratio of the eccentricity error and the inclination error in the total error. If *λ* < 10^−2^, the eccentricity error was two orders of magnitude smaller than the inclination error. In this case, the inclination error was the main source of the errors. In order to simplify the calculation, we could ignore the eccentricity error and use the inclination error to approximate the total error. If *λ* > 10^2^, the eccentricity error was two orders of magnitude larger than the inclination error. In this case, the eccentricity error was the main source of the errors. In order to simplify the calculation, we could ignore the inclination error and use the eccentricity error to approximate the total error.

Through the above-mentioned comparative analysis, the following conclusions were drawn: both the eccentricity error and the inclination error were the main factors of the angle measurement error of the circular grating. In specific applications, the proportion of the two should be analyzed before appropriate simplification.

## 4. Error Calibration and Compensation Experiment

### 4.1. Experiment Setup

**Figure 7:**
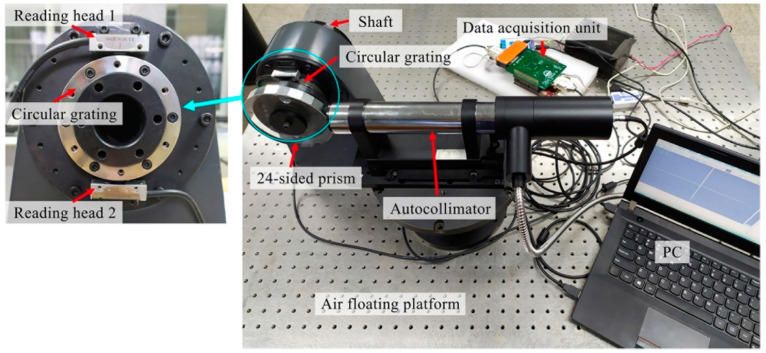
Experimental system for calibration and compensation of the angle measurement error of the circular grating.

The calibration and compensation experiment system for the angle measurement error of the circular grating included a shaft, a circular grating, an autocollimator, a 24-sided prism, a data acquisition unit, and an air floating platform, as shown in Figure 7. The angular measurement accuracy of the circular grating was 1.05″ and the resolution was 0.21″. The accuracy of the 24-sided prism was 0.5″. The accuracy of the Autocollimator was 0.5″ and the resolution was 0.01″. The sampling frequency of the data acquisition unit was 0.1 MHz. The air floating platform could isolate the vibration interference of the external environment. The 24-sided prism and the circular grating were installed on the same side of the shaft. The circular grating was equipped with double reading heads. The double reading heads were arranged on the upper and lower diameter positions of the circular grating. The parameters of the double reading heads were the same. When the shafting rotated to different positions, the data acquisition unit synchronously collected the angle measurement data of the autocollimator and the double reading heads. The measurement value of reading head one was used for the angle measurement error analysis of the single reading head. The synchronized measurement values of reading head one and reading head two were used for the measurement error analysis of the double reading heads. The error compensation method was described in detail in the next section.

### 4.2. Calibration and Compensation Method for the Angle Measurement Error

**Figure 8:**
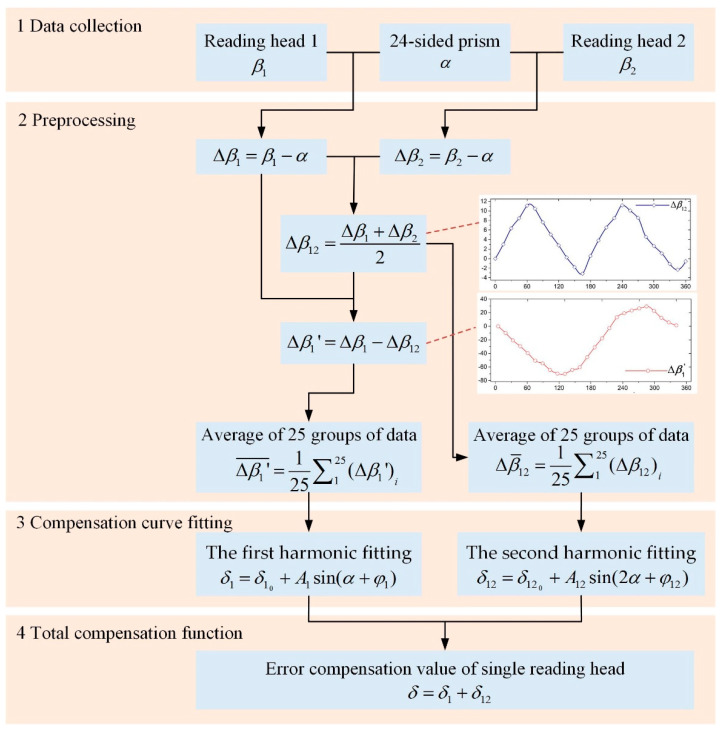
Calibration steps of the angle measurement error of the single reading head.

Before compensating the angle measurement error of the circular grating, the angle measurement system should be calibrated to obtain the compensation curve. The 24-sided prism and the circular grating were installed on the same side of the shaft. During the calibration experiment, we rotated the shaft so that the 24-sided prism was aligned with the autocollimator from the 0th side to the 23rd side in sequence. According to the characteristics of the 24-sided prism, the shafting rotated 15° each time, and the readings of the autocollimator and the double reading heads were recorded once. When the shafting rotated one circle, we obtained a set of measurement data. The above experiment was repeated 40 times to obtain 40 groups of measurement data. The 25 groups of measurement data were processed according to the calibration method steps in Figure 8 to obtain the angle measurement error compensation curve of a single reading head.

In Figure 8, after the shafting rotated one circle, the measurement value *β*_1_ of reading head one and the measurement value *β*_2_ of reading head two were, respectively, compared with the corresponding angle values of the 24-sided prism, and the angle measurement errors Δ*β*_1_ and Δ*β*_2_ were obtained. The arithmetic average of the two was Δ*β*_12_, which was the angle measurement error of the double reading heads. Through an observation, it was found that the discrete point distribution curve of Δ*β*_12_ was close to the second harmonic curve. According to the principle of eliminating the angle measurement error of the double reading heads, the uniform arrangement of the double reading heads could only eliminate odd harmonic error components. It shows that Δ*β*_12_ contained the second harmonic component. Then, the difference Δ*β*_1′_ between the angle measurement error Δ*β*_1_ of reading head one and the angle measurement error Δ*β*_12_ of the double reading heads had to include the angle measurement error described in Equation (21). The 25 groups of experimental data were processed to find the corresponding Δ*β*_12_ and Δ*β*_1′_, respectively. The arithmetic average was $\bar{\Delta \beta_{12}}$ and $\bar{\Delta \beta_{1}\prime }$. Performing a sine fitting on $\bar{\Delta \beta_{12}}$ and $\bar{\Delta \beta_{1}\prime }$, respectively, we obtained the second harmonic fitting error compensation function and the first harmonic fitting error compensation function.

The first harmonic fitting error compensation function was:

(22) $$\delta_{1}=\delta_{1_{0}}+A_{1}\sin(\alpha +\varphi_{1})$$

where $\varphi_{1}$ is the initial phase of the first harmonic, *A*_1_ is the first harmonic amplitude, and $\delta_{1_{0}}$ is the first harmonic amplitude offset. Comparing Equation (22) and Equation (21), it can be seen that the first harmonic fitting error compensation function was consistent with the total error model of the circular grating angle measurement.

The second harmonic fitting error compensation function was:

(23) $$\delta_{12}=\delta_{12_{0}}+A_{12}\sin(2\alpha +\varphi_{12})$$

where *α* is the true value of the rotation angle, $\varphi_{12}$ is the initial phase of the second harmonic, *A*_12_ is the second harmonic amplitude and $\delta_{12_{0}}$ is the second harmonic amplitude offset. Then, *δ* = *δ*_1_ + *δ*_12_ was the error compensation function of the single reading head, and Equation (23) was the error compensation function of the double reading heads.

According to the initial phases $\varphi_{1}$ and $\varphi_{12}$ of the fitting function and the position of the shaft corresponding to the 0th side of the prism, the initial flag of the error compensation could be determined. Then, in the experiment, according to the error compensation functions, starting from the initial flag, the angle measurement error of one revolution of the shafting could be compensated. To each group of measurement data, the compensation function *β*_1_* and the residual error *v*_1_ after the compensation of reading head one were, respectively:

(24) $$\beta_{1}*=\alpha +\delta$$

(25) $$v_{1}=\beta_{1}-\beta_{1}*$$

The compensation function *β*_12_* and the residual error *v*_12_ after the compensation of the double reading heads were, respectively:

(26) $$\beta_{12}*=\alpha +\delta_{12}$$

(27) $$v_{12}=\beta_{12}-\beta_{12}*$$

where *β*_12_ is the mean value of the double reading heads.

Using the remaining 15 groups of measurement data, we could calculate the residual error *v*_1_ of reading head one and the residual error *v*_12_ of the double reading heads according to the above equations, and validate the proposed error compensation functions.

## 5. Results and Discussion

### 5.1. Experimental Results and Discussion of the Angle Measurement Error

According to Section 4.2, the experiment was repeated 40 times to obtain 40 groups of angle measurement data. In total, 25 groups of measurement data were processed according to the steps shown in Figure 8. *β*_1_ and *β*_2_ in the fifth group of data, and the corresponding Δ*β*_1′_ and Δ*β*_12_, are listed in Table 5.

After averaging 25 groups of data, the data were fitted to obtain the parameter values in the compensation function of the angle measurement error of reading head one, as shown in Table 6.

According to the fitting results in Table 6, the compensation vectors ***δ***_1_, ***δ***_12,_ and ***δ*** could be obtained. The compensation results of the single reading head and the double reading heads are shown in Table 7.

**Figure 9:**
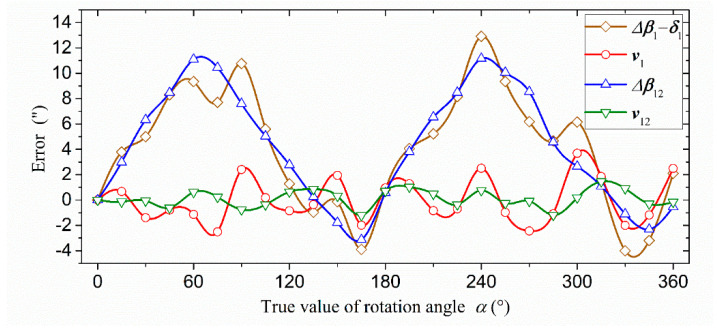
The error curve of reading head one and the error curve of the average value of the double reading heads.

In Table 7, the peak-to-peak error (16.92″) after the first harmonic compensation of reading head one was reduced to 17.05% of the peak-to-peak error (99.24″) before compensation, which was basically the same as the error (14.29″) of the mean value of the double reading heads. Further, the peak-to-peak error (6.19″) after the second harmonic compensation of reading head one was reduced to 6.24% of the peak-to-peak error (99.24″) before compensation, which was of the same orders of magnitude as the error (2.68″) after the second harmonic compensation of the mean value of the double reading heads. After two progressive compensations, the peak-to-peak error of reading head one was reduced by 93.76%, which was almost two orders of magnitude, and the compensation effect was very significant. The error curve of reading head one after compensation and the error curve of the average value of the double reading head are shown in Figure 9.

It can be intuitively seen in Figure 9 that the trend and amplitude of the error curve after the first harmonic compensation of reading head one and the error curve of the mean value of the double reading heads were basically the same. After the second harmonic compensation, the trend and amplitude of the error curve of reading head one were also very close to the error curve of the mean value of the double reading heads. The experimental results showed that the compensated angle measurement accuracy of the single reading head was consistent with that of the double reading heads. Moreover, comparing the data of *v*_1_ and Δ*β*_12_, we could also see that the error of the single reading head after two progressive compensations was much smaller than the error of the mean value of the double reading heads without compensation. It showed that the angle measurement accuracy of the single reading head after the secondary compensation was higher than the angle measurement accuracy of the mean value of the double reading heads without compensation.

**Figure 10:**
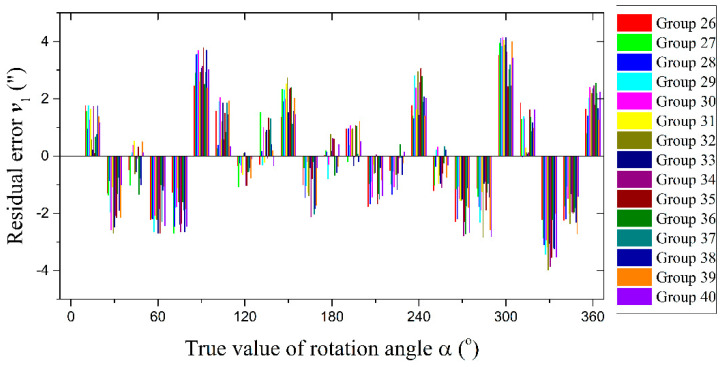
The residual error ***v***_1_ of reading head one after two progressive compensations (15 groups of measurement data).

Next, we used the remaining 15 groups of measurement data to verify the error compensation function obtained from the previous 25 groups of measurement data. The residual error ***v***_1_ of reading head one after two progressive compensations is shown in Figure 10.

The peak-to-peak value and uncertainty of residual errors in 15 groups (as shown in Figure 10) could be obtained. In the calculated results, the maximum value of the peak-to-peak value was 7.88″, and the maximum value of the uncertainty was 2.08″. The results showed that the measurement data had good repeatability, and the error compensation functions obtained by the method in Figure 8 could effectively compensate the angle measurement error of the circular grating and improve the angle measurement accuracy of the shafting.

### 5.2. Simulation Results of the Error Compensation in the Dynamic Torque Calibration System

For dynamic torque calibration, the shafting swung sinusoidally. The swing angle was set as *α*, and:

(28)  α=Asin(ωt+φ)

where *A* is the swing angle amplitude, φ is the phase, *ω* is the angular frequency, and *t* is the time. Then, the angular velocity was:

(29) $$\;\frac{d\alpha }{dt}=A\omega \cos(\omega t+\varphi )$$

The angular acceleration was:

(30) $$\frac{d^{2}\alpha }{dt^{2}}=-A\omega^{2}\sin(\omega t+\varphi )\;$$

When there were eccentricity and inclination errors in the angle measurement of the shafting, according to the method in Figure 8, the angle measurement compensation function could be obtained as shown in Equation (24). Substituting Equation (22) and Equation (23) into Equation (24), we obtained:

(31) $$\beta_{1}*=\alpha +\delta_{1_{0}}+A_{1}\sin(\alpha +\varphi_{1})+\delta_{12_{0}}+A_{12}\sin(2\alpha +\varphi_{12})$$

The derivative of *β*_1_* was:

(32) $$\frac{d\beta_{1}*}{dt}=\frac{d\alpha }{dt}(1+A_{1}\cos(\alpha +\varphi_{1})+2A_{12}\cos(2\alpha +\varphi_{12}))$$

Then, the second derivative of *β*_1_* was:

(33) $$\begin{array}{c} \frac{d^{2}\beta_{1}*}{dt^{2}}=\frac{d^{2}\alpha }{dt^{2}}(1+A_{1}\cos(\alpha +\varphi_{1})+2A_{12}\cos(2\alpha +\varphi_{12}))\\ -{\left(\frac{d\alpha }{dt}\right)}^{2}(A_{1}\sin(\alpha +\varphi_{1})+4A_{12}\sin(2\alpha +\varphi_{12})) \end{array}$$

Therefore, the error compensation function of the angular acceleration was as follows:

(34) $$\begin{array}{cc} \;\Delta \ddot{\beta } & =\frac{d^{2}\beta_{1}*}{dt^{2}}-\frac{d^{2}\alpha }{dt^{2}}\\ & =-A\omega^{2}\sin(\omega t+\varphi )(A_{1}\cos(\alpha +\varphi_{1})+2A_{12}\cos(2\alpha +\varphi_{12}))\\ & -A\omega \cos(\omega t+\varphi )(A_{1}\sin(\alpha +\varphi_{1})+4A_{12}\sin(2\alpha +\varphi_{12})) \end{array}$$

where $\Delta \ddot{\beta }$ is the error compensation value of the angular acceleration. Thus, according to Equation (1), the dynamic torque error was:

(35) $$\;\Delta T=J\Delta \ddot{\beta }$$

**Figure 11:**
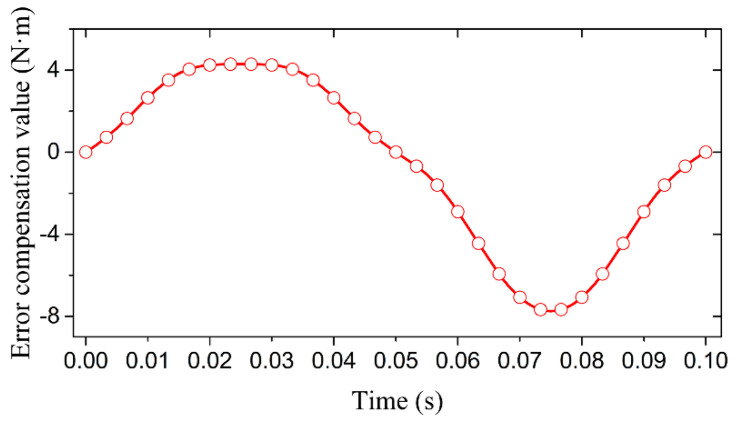
Error compensation curve of the dynamic torque.

In the simulation, let the swing angle amplitude be 90°, the frequency be 10 Hz, and the initial phase be 0°. Then, $\;\alpha =\frac{\pi }{2}\sin(20\pi t)$. Let the moment of inertia *J* be 2 kg·m^2^ and, using the compensation data in Table 6, we could obtain the dynamic torque compensation curve as shown in Figure 11. In Figure 11, the peak-to-peak value of the torque error compensation curve was 11.96 N·m. The simulation results showed that the application of the error compensation method shown in Figure 8 could improve the accuracy of the dynamic torque calibration system.

## 6. Conclusions

After analyzing the sources of the angle measurement error of the shafting, an eccentricity error model and an inclination error model were established, respectively, in this paper. Further, a total error model combining the two models was established. Through a simulation study with the models, the conditions, in which the eccentricity error or inclination error could be ignored, were discussed. The calibration and compensation methods of the angle measurement error were given, and the progressive error compensation function which integrated the first harmonic fitting and the second harmonic fitting was obtained. Then, an experiment was performed to verify the proposed calibration and compensation methods. Finally, the influence of the angle measurement error on the dynamic torque calibration system was simulated.

According to the experimental results, the peak-to-peak error of the single reading head after the first harmonic compensation was only about 17.05% of the peak-to-peak error before compensation, which was essentially the same as the mean value of the double reading heads. Further, the peak-to-peak error after the second harmonic compensation of the single reading head was only 6.24% of the peak-to-peak error before compensation, which was also very close to the error after the second harmonic compensation of the mean value of the double reading heads. After two progressive compensations, the peak-to-peak value of the compensated angle measurement error of the single reading head could be reduced by about 93.76%. In the verification experiment, the peak-to-peak value and uncertainty of residual errors in 15 groups could be obtained. In the calculated results, the maximum value of the peak-to-peak value was 7.88″ and the maximum value of the uncertainty was 2.08″. The experimental results showed that the error calibration and compensation method based on the proposed error model could effectively compensate the angle measurement error of the circular grating with a single reading head, and obtain a high-precision measurement angle.

The residual error after the second harmonic compensation also contained many other factors, mainly including the measurement error caused by the roundness and deformation of the circular grating, and the reading error caused by the reading head itself, etc. In the next work, we will conduct more in-depth research to obtain a higher angle measurement accuracy.

## Figures and Tables

**Table 1:** The coordinates of points before and after rotation.

	FCS Origin	RCS Origin	Measuring Point
Before rotation	*O*(0, 0)	*O′*(*x_O′_*, *y_O′_*)	*A*(*x_A_*, *y_A_*)
After rotation	*O*(0, 0)	*O″*(*x_O″_*, *y_O″_*)	*B*(*x_B_*, *y_B_*)

**Table 2:** Simulation data of the eccentricity error (unit: degree).

	*θ_e_*	0	15	30	45	90	180	270
*α*								
**0**		0	0	0	0	0	0	0
**15**		−0.002	0.002	0.006	0.009	0.015	0.002	−0.015
**30**		−0.008	0	0.008	0.015	0.029	0.008	−0.029
**45**		−0.017	−0.006	0.006	0.017	0.041	0.017	−0.041
**90**		−0.057	−0.041	−0.021	0	0.057	0.057	−0.057
**180**		−0.115	−0.111	−0.099	−0.081	0	0.115	0.000
**270**		−0.057	−0.070	−0.078	−0.081	−0.057	0.057	0.057

**Table 3:** Simulation data of the inclination error (unit: degree).

	*θ_L_*	0	15	30	45	90	180	270
*α*								
**0**		0	0	0	0	0	0	0
**15**		−0.009	0.007	−0.002	−0.005	0.004	0.005	−0.009
**30**		−0.018	0.013	−0.001	−0.011	0.006	0.013	−0.018
**45**		−0.026	0.016	0.002	−0.019	0.006	0.021	−0.025
**90**		−0.041	0.012	0.023	−0.046	−0.008	0.048	−0.035
**180**		−0.011	−0.037	0.068	−0.065	−0.058	0.063	0.002
**270**		0.030	−0.049	0.045	−0.019	−0.050	0.015	0.036

**Table 4:** Simulation data of the total error (unit: degree).

	*θ_e_*	0	15	30	45	90	180	270
*α*								
**0**		0	0	0	0	0	0	0
**15**		−0.011	0.009	0.004	0.004	0.019	0.007	−0.024
**30**		−0.026	0.013	0.007	0.004	0.035	0.020	−0.046
**45**		−0.043	0.010	0.008	−0.003	0.047	0.038	−0.065
**90**		−0.098	−0.029	0.002	−0.046	0.049	0.105	−0.092
**180**		−0.126	−0.148	−0.032	−0.146	−0.058	0.177	0.002
**270**		−0.028	−0.119	−0.034	−0.100	−0.107	0.072	0.094

**Table 5:** The fifth group of angle measurement data and errors.

*α* (°)	**15**	**30**	**45**	**60**	**75**	**90**	**105**	**120**
*β*_1_ (°)	14.9972	29.9942	44.9918	59.9890	74.9859	89.9848	104.9821	119.9806
*β*_2_ (°)	15.0044	30.0093	45.0129	60.0172	75.0199	90.0194	105.0207	120.0209
Δ*β*_1′_ (″)	−12.89	−27.20	−38.08	−50.78	−61.19	−62.39	−69.31	−72.57
Δ*β*_12′_ (″)	2.98	6.33	8.47	11.10	10.45	7.60	5.04	2.78
*α* (°)	**135**	**150**	**165**	**180**	**195**	**210**	**225**	**240**
*β*_1_ (°)	134.9805	149.9821	164.9832	179.9873	194.9914	209.9951	224.9992	240.0037
*β*_2_ (°)	135.0196	150.0169	165.0150	180.0130	195.0107	210.0086	225.0055	240.0025
Δ*β*_1′_ (″)	−70.33	−62.49	−57.20	−46.30	−34.89	−24.31	−11.25	2.03
Δ*β*_12′_ (″)	0.25	−1.78	−3.13	0.57	3.81	6.54	8.49	11.16
*α* (°)	**255**	**270**	**285**	**300**	**315**	**330**	**345**	**360**
*β*_1_ (°)	255.0053	270.0065	285.0073	300.0081	315.0063	330.0035	345.0016	360.0003
*β*_2_ (°)	255.0003	269.9983	284.9952	299.9934	314.9943	329.9959	344.9971	359.9994
Δ*β*_1′_ (″)	9.11	14.74	21.70	26.50	21.63	13.53	8.00	1.77
Δ*β*_12′_ (″)	10.07	8.54	4.56	2.65	1.10	−1.10	−2.31	−0.55

**Table 6:** The parameter values in the angle measurement error compensation function of reading head one.

Parameter	$\varphi_{1}\;({}^{\circ})$	$\varphi_{12}\;({}^{\circ})$	*A*_1_ (″)	*A*_12_ (″)	$\delta_{1_{0}}$ (″)	$\delta_{12_{0}}$ (″)
**Value**	208.711	19.791	47.05	6.40	−24.04	4.18

**Table 7:** Comparison of the peak-to-peak error between reading head one and the double reading heads (unit: arc second).

	Peak-to-Peak Error before Compensation	Peak-to-Peak Error after First Harmonic Compensation	Peak-to-Peak Error after Second Harmonic Compensation
**Reading Head One**	Δ*β*_1_	Δ*β*_1_ − *δ*_1_	*v*_1_
	99.24	16.92	6.19
**Double Reading Heads**	Δ*β*_12_	-	*v*_12_
	14.29	-	2.68
